# Despite Genetic Iron Overload, *Hfe*‐Hemochromatosis Mice Do Not Show Bone Loss

**DOI:** 10.1002/jbm4.10206

**Published:** 2019-07-26

**Authors:** Alessa Wagner, Betül Alan, Dilay Yilmaz, Mubashir Ahmad, Peng Liu, Naveen Kumar Tangudu, Jan P Tuckermann, Maja Vujic Spasic

**Affiliations:** ^1^ Institute of Comparative Molecular Endocrinology, University of Ulm Ulm Germany

**Keywords:** HFE‐HEMOCHROMATOSIS, IRON, BONE, OSTEOPOROSIS, OSTEOBLAST/OSTEOCLASTS

## Abstract

One of the most prevalent genetic iron overload disorders in Caucasians is caused by mutations in the *HFE* gene. Both *HFE* patients and *Hfe*‐mouse models develop a progressive accumulation of iron in the parenchymal cells of various tissues, eventually resulting in liver cirrhosis, hepatocellular carcinoma, cardiomyopathies, hypogonadism, and other pathologies. Clinical data and preclinical models have brought considerable attention to the correlation between iron overload and the development of osteoporosis in *HFE/Hfe* hemochromatosis. Our study critically challenges this concept. We show that systemic iron overload, at the degree present in *Hfe* ^−/−^ mice, does not associate with the microarchitecture impairment of long bones, thus excluding a negative effect of iron overload on bone integrity. We further reveal that Hfe actions in osteoblasts and osteoclasts are dispensable for the maintenance of bone and iron homeostasis in mice under steady‐state conditions. We conclude that, despite systemic iron overload, *Hfe* ^−/−^ mice present normal physiological bone homeostasis. © 2019 The Authors. *JBMR Plus* in published by Wiley Periodicals, Inc. on behalf of the American Society for Bone and Mineral Research.

## Introduction

Disorders of iron metabolism, such as iron deficiency^1^ and acquired and genetic iron overload,^2^ constitute an increasing public health problem worldwide. Hereditary hemochromatosis (HH) is a genetic iron overload disorder characterized by excessive iron absorption from the diet and increased iron deposition in tissues.^2^ The most prevalent form of HH in a population of northern European ancestry is caused by mutations in the *HFE* gene with a carrier frequency of approximately one in eight.^3^, ^4^ The disease is incompletely penetrant and affects men more severely than women.^5^, ^6^ Mutations in the *HFE/Hfe* gene result in low production of hepcidin, a small liver‐derived peptide hormone,^7^ which in turn acts to increase iron absorption from the diet and iron release from enterocytes and macrophages into the circulation.^8^

Studies in *HFE*‐HH patients and respective disease animal models showed that HFE/Hfe actions in hepatocytes were essential to maintain appropriate hepcidin expression and systemic iron levels, establishing *Hfe*‐HH as the liver disease.^9^, ^10^, ^11^ At the molecular level, the lack of *Hfe* impaired the activity of the cytoplasmic receptor‐activated small mothers against the decapentaplegic (R‐Smad) signaling pathway, resulting in low hepcidin expression.^12^, ^13^, ^14^, ^15^ Similarly, mice with a genetic disruption of both *Hfe* and *TfR2*,^16^ bone morphogenitic protein (Bmp) coreceptor hemojuvelin (*Hjv*)^17^ or *Bmp2*,^18^ *Bmp6*,^19^, ^20^, ^21^ hepatic Bmp‐receptors type I (*Alk3*, *Alk2*)^22^ or *Smad4*,^23^ showed impaired Smad signaling and low hepatic hepcidin expression and consequently developed HH phenotype, demonstrating the critical importance of the iron‐regulated Bmp/Smad signaling pathway for hepcidin regulation.

However, because there is no regulated mechanism to remove the excess of iron, a progressive iron accumulation in various tissues occurs, resulting in multiple organ dysfunction and eventually failure. Liver cirrhosis, hepatocellular carcinoma, cardiomyopathies, and hypogonadism are commonly described in patients with iron‐overload disorders.^2^ Clinical data have drawn considerable attention to the strong correlation between iron overload and the development of osteoporosis in disorders such as congenital and acquired anemias (eg, thalassemias, sickle cell disease).^24^, ^25^, ^26^ Moreover, osteoporosis was initially described as a frequent complication in *HFE*‐HH patients; however, most patients had evidence of gonadal hormone deficiency, which is likely the primary effect of iron on reported bone loss.^27^, ^28^ Further study reported a significant bone loss in 26% of *HFE‐*hemochromatosis patients, which positively associated with increased levels of total alkaline phophatase, the presence of hypogonadism, and the severity of iron overload, comorbidities that can account for secondary osteoporosis.^29^ Interestingly, lower BMD was described in 34% of men with genetic *HFE*‐HH, which was not related with hyperparathyroidism, vitamin D deficiency, hypogonadism, or cirrhosis.^30^ Subsequent studies in *Hfe* ^−/−^ mice proposed iron overload as the main cause of bone loss in *Hfe*‐HH^31^, ^32^; however, some inconsistencies between the data exist as to whether iron overload affects the bone integrity by increasing osteoclastogenesis or by impairing bone formation.

Considering these data, it is surprising that iron‐depletion therapies (ie, phlebotomies, desferrioxamine) that are remarkably successful in preventing liver and heart complications do not fully improve bone quality.^33^, ^34^ The adverse effects of phlebotomies describing increased cartilage degradation have been reported in a number of venesected patients.^33^ Depleting iron levels with desferrioxamine will result in systemic iron deficiency, which is likely to suppress bone resorption; on the other hand, it will also inhibit bone formation, as previously reported in rodent models.^34^ Moreover, the increased expression and release of certain inflammatory mediators have been associated with desferrioxamine therapies.^35^

Given these studies, we aimed to dissect whether direct functions of Hfe in osteoblasts and osteoclasts were decisive for the control of bone integrity or whether iron overload, caused by *Hfe* impairment, led to bone loss. Our data unequivocally showed that neither the lack of *Hfe* in bone cells nor the iron overload at the levels present in *Hfe* ^−/−^ mice, suffice to trigger bone loss, thus critically challenging the concept of iron‐mediated bone loss in *Hfe*‐HH.

## Materials and Methods

### Mice

*Hfe* ^−/−^ mice, both males and females at the ages of 11, 29, and 53 weeks, and their littermate controls (*Hfe* ^+/+^), maintained on a C57BL/6 J genetic background, were used in this study. A new mouse line lacking *Hfe* in osteoblasts, the *Hfe* ^*Runx2Cre*^, was generated by crossing *Hfe* ^*flox*^ mice with *Runx*2*Cre* transgenic mice expressing Cre recombinase under the control of the bone‐specific distal promoter of the *Runx*2 gene^36^ (Supplementary Fig. 1 *A*). Mice with hepatocyte‐ and osteoclast‐specific *Hfe* deletion (*Hfe* ^*AlfpCre(+)*^ and *Hfe* ^*LysMCre(+)*^ mutant mice, respectively) have been previously described.^10^ All mice received two intraperitoneal calcein injections (100 µL of a 10 mg/mL solution; Sigma‐Aldrich, St. Louis, MO, USA). Mice were kept under a standard mouse diet containing 180 mg/kg iron (Ssniff, Soest, Germany). Mice were euthanized by CO_2_ inhalation. Blood, livers, and the bones were collected. Animal experiments were approved and performed in accordance with the University Animal Care Committee and the Federal Authorities for Animal Research (Regierungspraesidium Tuebingen, Baden‐Wuerttemberg, Germany).

### Micro‐CT analysis

Femurs and spins were collected and fixed in 4% paraformaldehyde (Merck, Darmstadt, Germany) for 3 days and stored in 0.5% paraformaldehyde until further analysis. Distal regions of right femur and lumbar vertebra were subjected to 3D µCT analysis using SkyScan 1174 compact in vitro µCT or SkyScan 1176 in vivo high‐resolution µCT (Bruker, Billerica, MA, USA) equipped with an X‐ray tube with a voltage of 50 to 80 kV/100 μA, using a 6‐μm voxel size and 0.5‐degree rotation step. The fifth lumbar vertebras were measured at 9 µm and the rotation step was set at 1 degree. To reduce beam hardening, a 0.5‐mm aluminium filter was used. For the reconstruction of femora, the trabecular ROI was selected to extend 0.3 mm proximally to the end of the distal growth plate over 1.8 mm towards the diaphysis. Images of femora were acquired. The morphometric parameters defining trabecular bone mass, including trabecular bone volume/tissue volume (BV/TV), trabecular thickness (Tb.Th), trabecular separation (Tb.Sp), and trabecular number (Tb.N), were analyzed at the femoral midshaft according to the guidelines issued by the ASBMR Histomorphometry Nomenclature Committee.^37^

### Bone histomorphometry

Static and dynamic histomorphometry was performed on decalcified and undecalcified femoral sections of mice.

For static analysis, femurs or tibias were fixed in 4% paraformaldehyde (Merck) for 3 days, washed for 2 hours with water, and decalcified in the presence of 15% EDTA for 10 days with mild shaking at 37°C. The bones were embedded in paraffin. Osteoclast number/bone perimeter and osteoclast surface/bone surface were quantified on decalcified sections of either tibia or femur stained for tartrate‐resistant acid phosphatase (TRAP).

Undecalcified femurs were first dehydrated and infiltrated with destabilized methylmetacrylate (Merck), benzoylperoxide (Merck), and nonylphenyl‐polyethyleneglycol acetate (Sigma‐Aldrich, St. Louis, MO, USA) for 14 days. Embedding in methylmetracrylate was done overnight at 4°C, and the femurs were used for dynamic analysis. Osteoblast number/bone perimeter, osteoblast surface/bone surface, and osteocyte number were quantified on toluidine blue‐stained undecalcified femoral sections. Two sections were analyzed for each mouse. Bone formation rate (BFR)/bone surface was measured on undecalcified bone sections by means of fluorochrome labeling. Osteomeasure software was used for the analysis (Osteometrics, Decatur, GA, USA)

### Immunohistochemistry

Immunohistochemical analysis of osteocalcin was performed on paraffin‐embedded femur tissues (5 µm). Briefly, sections were deparaffinized and blocked with 10% goat serum for 2 hours at 4°C, followed by incubation with monoclonal antiosteocalcin antibody (1:500; ab93876; Abcam, Cambridge, MA, USA) overnight at 4°C. Thereafter, sections were incubated in 0.3% H_2_O_2_ (Merck) for 15 min, blocked with Avidin/Biotin Blocking Kit (15 min/15 min; Biozol, Bayern, Germany) and incubated with secondary antibody (biotynliated antirabbit, 1:800; BA‐1000; Vector Laboratories, Burlingame, CA, USA) for 2 hours at 4°C. Afterwards, sections were incubated with horseradish peroxidase streptavidin (1:300; SA‐5004; Vector Laboratories) for 60 min at 4°C and stained with ImmPACT DAB Peroxidase Substrate Kit (Vector Laboratories) for 3 min. Counterstaining was done with Hematoxylin Gill's Formula (Vector Laboratories) for 5 min; dehydration slides were mounted with Eukitt Mounting Media (R. Langenbrick, Emmendingen, Germany). Sections were visualized under a microscope (Olympus, Hamburg, Germany) using 400 × magnification. Sections without primary or secondary antibody were also included. Cells positive for osteocalcin stained brown.

### Iron measurement and staining

The nonheme iron content in the liver was measured as previously described.^10^ Plasma iron was determined with an iron kit (Thermo Fisher Scientific, Vantaa, Finland) in a 96‐well format using a serial dilution of iron atomic absorption standard solution (1000 mg/mL iron in HCl; Sigma‐Aldrich, St. Louis, MO, USA) according to the manufacturer's instructions. Iron values are expressed as µg of iron per dL.

Prussian blue staining was used to visualize iron deposition on livers and bones. Livers were paraffin‐embedded and cut in 4‐µm thick sections. Iron staining of bones was performed on both decalcified and undecalcified bones. Bones were first fixed in 4% paraformaldehyde (Merck) for 3 days and washed for 2 hours with water. Afterwards, bones were decalcified in 15% EDTA for 10 days with mild shaking at 37°C. The EDTA solution was changed every third day. Decalcified and paraffin‐embedded femurs (5‐µm sections) were mounted on polylysine‐coated slides (R. Langenbrick). Undecalcified femurs were first dehydrated and infiltrated with destabilized methylmetacrylate (Merck), benzoylperoxide (Merck), and nonylphenyl‐polyethyleneglycol acetate (Sigma‐Aldrich, St. Louis, MO, USA) for 14 days. Embedding in methylmetracrylate was done overnight at 4°C. Undecalcified and plastic‐embedded femurs were mounted on superforst slides (R. Langenbrick).

All sections were stained for iron in the presence of 1% HCl and 2% potassiumhexacyanoferrate II trihydrate (Merck) for 60 min. Nucleus was counterstained with Nuclear Fast Red Solution (Sigma‐Aldrich, St. Louis, MO, USA) for 10 min and slides were mounted with Eukitt Mounting Media (R. Langenbrick). Sections were visualized under a microscope (Olympus) using 40× (liver) and 100× (bone) magnification.

Quantification of iron staining in the bones was performed on undecalcified femur sections. Iron‐labeled surface per bone surface was determined using Osteomeasure software (Osteometrics).

### Enzyme‐linked immunosorbent assay

P1NP and C‐terminal collagen crosslinks telopeptide 1 (CTX‐I), markers of bone formation and resorption, respectively, were measured in plasma using ELISA kits, according to the manufacturer's instructions (Immunodiagnostic Systems, Boldon Business Park, UK). Testosterone levels were measured in the plasma using an ELISA kit according to the manufacturer's instructions (DRG Diagnostics, Marburg, Germany).

### Isolation and differentiation of calvarial osteoblasts

Primary osteoblasts were isolated from neonatal mouse calvarias as previously described.^38^ *Hfe* ^*Runx2Cre*^, *Hfe* ^*−/−*^, and *Hfe* ^*AlfpCre*^ mice, 3 to 5 days old, were used. Briefly, calvarias were digested in Gibco Minimum Essential Medium Alpha (α‐MEM; Thermo Fisher Scientific, Bremen, Germany) containing 0.2% collagenase A (Sigma‐Aldrich C9891; Sigma‐Aldrich, Darmstadt, Germany), 0.2% dispase II (Boehringer Mannheim 165859; Boehringer Mannheim, Mannhaim, Germany) at 37°C for 10 min at 700 rpm. After centrifugation at 1500 rpm for 5 min, cells were seeded in 6‐well plates. Primary cells were passaged once, and osteogenic differentiation was induced by adding 100 mg/mL ascorbic acid (Sigma‐Aldrich, Darmstadt, Germany) and 5 mM beta‐glycerophosphate (Sigma‐Aldrich, Darmstadt, Germany) for 6 days.

For the determination of differentiation, after 6 days PFA‐fixed osteoblasts were stained for alkaline phosphatase (ALP) using Fast Violet B Salt (Sigma‐Aldrich, Darmstadt, Germany) and naphtol AS‐MS phosphate alkaline solution (Sigma‐Aldrich, Darmstadt, Germany). Images were captured using a Canon EOS 600D camera (Canon, Tokyo, Japan). For quantification, the Amplite Colorimetric Alkaline Phosphatase Assay Kit (11950; AAT Bioquest, Sunnyvale, CA, USA) was used according to the manufacturer's instructions. ALP activity was normalized by cell viability via Presto Blue Assay (Invitrogen, Bremen, Germany).

### Differentiation of primary osteoclasts and TRAP staining

Bone marrows were isolated from the femora of 2‐ to 3‐month‐old *Hfe* ^*LysMCre*^ mice; 1 million cells were seeded per well in 24‐well plates. Differentiation of osteoclasts was performed in α‐MEM medium containing 10% FBS and 1% penicillin/streptomycin, supplemented with 50 ng/mL RANKL (PeproTech, Rocky Hill, NJ, USA) and 25 ng/mL M‐CSF (R&D Systems, Minneapolis, MN, USA) for 5 or 7 days. TRAP staining was performed using a TRAP Kit (Sigma‐Aldrich, Darmstadt, Germany).

### RNA isolation, reverse‐transcription, and real‐time PCR

Total RNA was isolated from primary osteoblasts using RNeasy Mini Kit (QIAGEN, Hilden, Germany) according to the manufacturer's instructions. RNA quality and quantity were controlled using the Nanodrop 2000 system (Thermo Scientific, Waltham, MA, USA). RevertAid H Minus Reverse Transcriptase (Fermentas, Waltham, MA, USA), 5× RT reaction buffer (Fermentas), random primers (200 ng/µL; Invitrogen, Carlsbad, CA, USA), and 10 mM dNTPs (Bioline, London, UK), were used to convert 2 µg of RNA to cDNA following the manufacturer's instructions. Quantitative real‐time PCR (qRT‐PCR) was carried out in 10 µL of reaction volume using SYBR Green I Dye (Invitrogen, Carlsbad, CA, USA) on ABI ViiA‐7 system (Applied Biosystems, Foster City, CA, USA). The mRNA abundance of the gene of interest was calculated relative to the expression of the reference gene *Gapdh* using the ∆∆CT method.^39^ Primers used in the study were:

**Table:** 

Primer name	Forward sequence (5’‐3’)	Reverse sequence (5’‐3’)
*Alp*	GCTGATCATTCCCACGTTTT	CTGGGCCTGGTAGTTGTTGT
*Fpn*	TGTCAGCCTGCTGTTTGCAGGA	TCTTGCAGCAACTGTGTCACCG
*Gapdh*	CCCATTCTCGGCCTTGACTGT	GTGGAGATTGTTGCCATCAACGA
*Hamp*	ATACCAATGCAGAAGAGAAGG	AACAGATACCACACTGGGAA
*Hfe*	CACCGTCTGTGCCATCTTCTT	ACATAGCCACCCATGGTTCCT
*Runx2*	CCTGAACTCTGCACCAAGTCCT	TCATCTGGCTCAGATAGGAGGG
*Sp7*	CCCACCCTTCCCTCACTCAT	CCTTGTACCACGAGCCATAGG
*TfR1*	CCCATGACGTTGAATTGAACCT	GTAGTCTCCACGAGCGGAATA

### Protein isolation and Western blot analysis

Protein extracts were prepared from primary osteoblasts as previously described.^15^ Membranes were blotted with antitransferrin receptor 1 (Tfr1; 1:500; Zymed Laboratories, South San Francisco, CA, USA) and antiferritin‐H (1:1000 in 2% BSA; Cell Signaling Technology, Danvers, MA, USA) following the manufacturer's instructions. Membranes were washed and incubated with antirabbit or antimouse horseradish peroxidase‐conjugated antibody (1:5000; Invitrogen, Carlsbad, CA, USA). Reactions were carried out with Luminata Forte Western HRP Substrate Kit (MilliporeSigma, Burlington, MA, USA). As a loading control, anti‐β‐actin (1:10000; Sigma‐Aldrich, St. Louis, MO, USA) was used. Membranes were washed prior to the addition of substrate and visualized in a chemiluminescence detector (Bio‐Rad Laboratories, Hercules, CA, USA).

### Statistical analyses

Data were analyzed using GraphPad Prism software (GraphPad Software, La Jolla, CA, USA); results are shown as mean ± SD. For the statistical analysis, a nonparametric distribution and the Mann‐Whitney *U* test were used. Statistically significant differences are indicated as *p* < .05 (*), *p* < .01 (**), and *p* < .005 (***) in the tables.

## Results

To determine whether (1) direct functions of Hfe in the bone cells, or (2) an excess of iron at the levels present in *Hfe‐*HH mice trigger bone loss, we employed three conditional mouse lines in which *Hfe* was specifically deleted in osteoblasts (*Hfe* ^*Runx2Cre*^), and in myeloid cells, including osteoclasts (*Hfe* ^*LysMCre*^)^40^, ^41^ and hepatocytes (*Hfe* ^*AlfpCre*^).^10^ The *Hfe* ^*AlfpCre(+)*^ mutant mouse is a unique model of *Hfe*‐HH, which because of a lack of *Hfe* exclusively in its hepatocytes, develops iron overload and phenocopies the pathology of global *Hfe* ^−/−^ mice.^10^

### Hfe actions in bone cells are dispensable for normal physiological bone and iron homeostasis in mice

**Figure 1:**
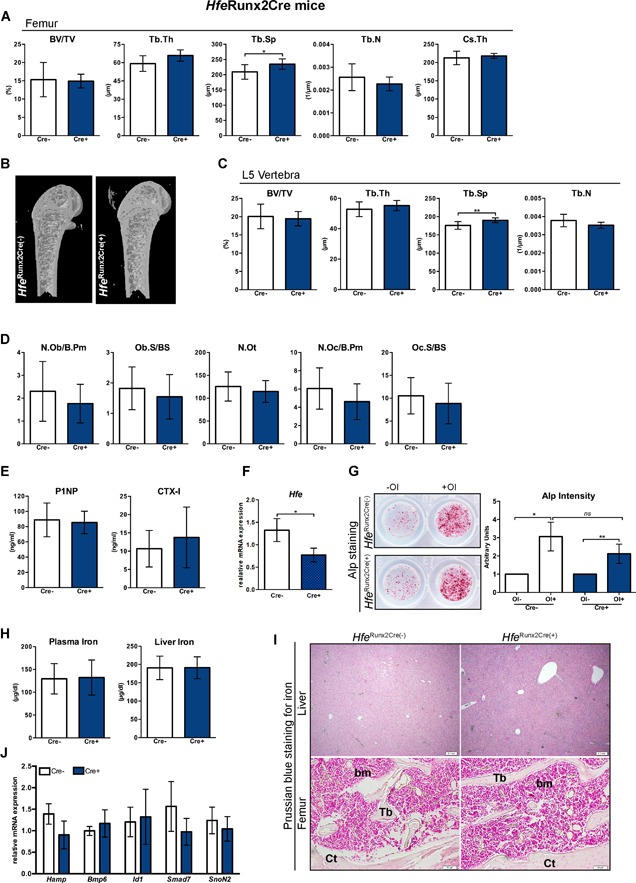
Hfe actions in osteoblasts are dispensable for the regulation of bone and iron metabolism. (*A, C*) µCT analysis of trabecular bone at distal femur and in the vertebra of *Hfe* ^*Runx2Cre(+)*^ and *Hfe* ^*Runx2Cre(−)*^ mice (*n* = 7; 10). (*B*) µCT 3D models of femora showing no evident changes in the composition of the trabecular bone, cortical bone, and marrow area. Images are representative of three mice per each group. (*D*) Histomorphometry showing no significant changes in osteoblast, osteocyte and osteoclast numbers between *Hfe* ^*Runx2Cre(+)*^ and *Hfe* ^*Runx2Cre(−)*^ mice (*n* = 6; 9). (*E*) The expression levels of bone formation marker P1NP, bone resorption marker C‐terminal collagen crosslinks telopeptide 1 (CTX‐I) in the serum of *Hfe* ^*Runx2Cre(+)*^ and *Hfe* ^*Runx2Cre(−)*^ mice (*n* = 6; 10). (*F*) Relative mRNA expression of *Hfe* in primary osteoblasts from *Hfe* ^*Runx2Cre(+)*^ and *Hfe* ^*Runx2Cre(−)*^ mice (*n* = 4; 5), measured by quantitative real‐time PCR (qRT‐PCR) and calculated relative to the expression of the reference gene *Gapdh*. (*G*) Alkaline phosphatase (ALP) staining and activity of primary osteoblasts from *Hfe* ^*Runx2Cre(+)*^ and *Hfe* ^*Runx2Cre(−)*^ mice cultured in the presence or absence of osteoblast differentiation medium (OI) for 7 days. ALP staining was performed at day 6. Representative images of primary osteoblasts derived from calvarias of neonatal *Hfe* ^*Runx2Cre(+)*^ and *Hfe* ^*Runx2Cre(−)*^ mice (*n* = 9; 9). ALP activity was measured in the supernatant at day 6 of osteoblast differentiation and was normalized to the cell viability. (*H*) Circulating iron levels and the nonheme liver iron content in *Hfe* ^*Runx2Cre(+)*^ and *Hfe* ^*Runx2Cre(−)*^ mice (*n* = 7; 10). (*I*) Prussian blue staining for iron depositions in the liver and femoral bone (decalcified) of *Hfe* ^*Runx2Cre(+)*^ and *Hfe* ^*Runx2Cre(−)*^ mice. Images are representative staining of three mice per each group. The pictures represent 40× (liver) and 100× (bone) magnification. (*J*) Relative mRNA expression of iron genes in the liver of *Hfe* ^*Runx2Cre(+)*^ and *Hfe* ^*Runx2Cre(−)*^ mice (*n* = 4; 5), measured by qRT‐PCR and calculated relative to the expression of the reference gene *Gapdh*. Data were analyzed using GraphPad Prism software and results are shown as mean ± SD. For the statistical analysis, a nonparametric distribution and the Mann‐Whitney *U* test were used. *p values < .05, **p values < .01. All mice were males 13 weeks of age. BV/TV = bone volume/tissue volume; Tb.Sp = trabecular separation; Tb.Th = trabecular thickness; Tb.N = trabecular number; Tb = trabecular bone; Ct = cortical bone; bm = bone marrow; N.Ob/B.Pm = osteoblast number/bone perimeter; Ob.S/BS = osteoblast surface/bone surface; N.Ot = osteocyte number; N.Oc/B.Pm = osteoclast number/bone perimeter; Oc.S/BS = osteoclast surface/bone surface; OI = osteogenic induction.

We first assessed the bone status in mice with selective *Hfe* deficiency in osteoblast (*Hfe* ^*Runx2Cre*^ mice). The analysis of cancellous bone parameters, such as BV/TV, the Tb.Th, the Tb.N, and the Cs.Th, revealed no statistically significant differences; however, minor differences were detected in the Tb.Sp in the femur and the vertebra of *Hfe* ^*Runx2Cre(+)*^ mutant mice when compared with sex/age‐matched *Hfe* ^*Runx2Cre(–)*^ littermate controls (Fig. 1 *A–C*). Moreover, the number of osteoblasts, osteoclasts, and osteocytes as well as the levels of serum P1NP and CTX‐I, markers of bone formation and bone resorption activity, respectively, were not significantly different between *Hfe* ^*Runx2Cre(+)*^ mutant mice and *Hfe* ^*Runx2Cre(−)*^ control littermates (Fig. 1 *D, E*). In line with these observations, the differentiation capacities of primary osteoblasts, isolated from neonatal calvaria from *Hfe* ^*Runx2Cre(+)*^ mutant and *Hfe* ^*Runx2Cre(−)*^ control, were not significantly different as evidenced by the measurement of the levels of alkaline phosphatase (Fig. 1 *F, G*). These data imply that trabecular microarchitectural organization is unaffected in *Hfe* ^*Runx2Cre(+)*^ mutant mice. With respect to iron status, we show that the lack of *Hfe* in the osteoblasts did not affect systemic iron metabolism because the levels of iron in the blood, liver, and the femur (Fig. 1 *H, I*), as well as the expression levels of major hepatic iron genes, such as hepcidin (*Hamp*), *Bmp6*, *Id1*, and *Smad7*, in *Hfe* ^*Runx2Cre(+)*^ mutant mice were similar to the levels in control mice (Fig. 1 *J*). Importantly, no overt changes in bone and iron parameters were detected in aged *Hfe* ^*Runx2Cre(+)*^ mutant mice (Supplementary Fig. 1).

**Figure 2:**
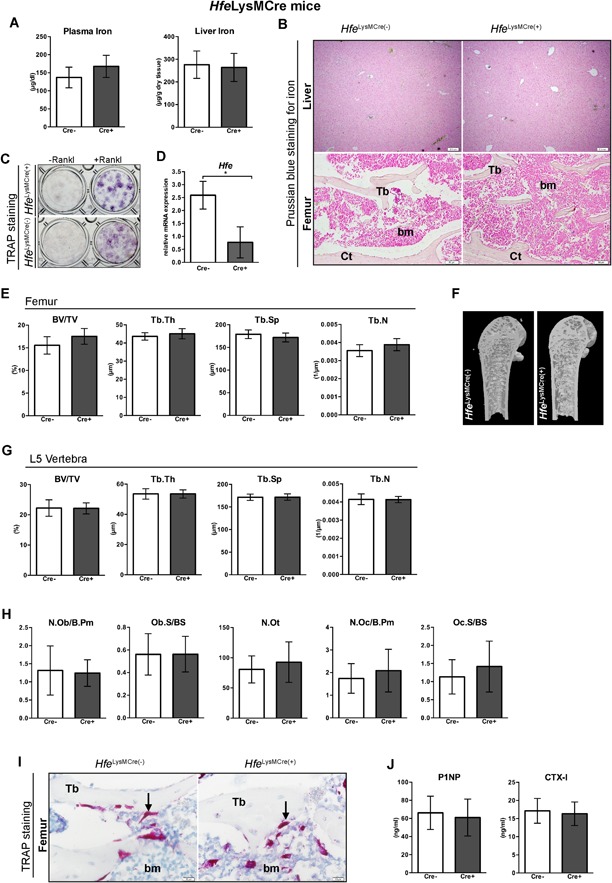
Hfe actions in osteoclasts are nonessential for the regulation of iron and bone metabolism. (*A*) Iron levels in the blood and the liver of *Hfe* ^*LysMCre(+)*^ and *Hfe* ^*LysMCre(−)*^ mice (*n* = 3; 6). (*B*) Prussian blue staining for iron depositions in the liver and femoral bone (decalcified) of *Hfe* ^*LysMCre(+)*^ and *Hfe* ^*LysMCre(−)*^. Images are representative staining of three mice per each group. The pictures represent 40× (liver) and 100× (bone) magnification. (*C*) Representative images of differentiated osteoclasts from *Hfe* ^*LysMCre(+)*^ and *Hfe* ^*LysMCre(−)*^ mice (*n* = 3; 3). (*D*) mRNA expression of *Hfe* in cultured osteoclasts from *Hfe* ^*LysMCre(+)*^ mutant mice measured by quantitative real‐time PCR and calculated relative to the expression of the reference gene *Gapdh* (*n* = 3; 4). (*E, G*) µCT analysis of trabecular bone at distal femur and in the vertebra of *Hfe* ^*LysMCre(+)*^ and *Hfe* ^*LysMCre(−)*^ mice (*n* = 6; 8). (*F*) µCT 3D models of femora showing no evident changes in the composition of the trabecular bone, cortical bone, and marrow area. Images are representative of three mice per each group. (*H*) Histomorphometry showing no significant changes in osteoblast, osteocyte, and osteoclast numbers between *Hfe* ^*LysMCre(+)*^ and *Hfe* ^*LysMCre(−)*^ mice (*n* = 4; 4). (*I*) TRAP‐positive cells in bone sections are indicated by an arrow and are stained red. Images are representative staining of three mice per each group. The pictures represent 400× magnification. (*J*) The expression levels of bone formation marker P1NP, and bone resorption marker C‐terminal collagen crosslinks telopeptide 1 (CTX‐I) in the serum of *Hfe* ^*LysMCre(+)*^ and *Hfe* ^*LysMCre(−)*^ mice (*n* = 6; 6). Data were analyzed using GraphPad Prism software and results are shown as mean ± SD. For the statistical analysis, a nonparametric distribution and the Mann‐Whitney *U* test were used. *p values < .05. All mice were males 13 weeks of age. BV/TV = bone volume/tissue volume; Tb.Sp = trabecular separation; Tb.Th = trabecular thickness; Tb.N = trabecular number; Tb = trabecular bone; Ct = cortical bone; bm = bone marrow; TRAP = tartrate‐resistant acid phosphatase; RANK‐L = receptor activator of NF‐κB ligand; N.Ob/B.Pm = osteoblast number/bone perimeter; Ob.S/BS = osteoblast surface/bone surface; N.Ot = osteocyte number; N.Oc/B.Pm = osteoclast number/bone perimeter; Oc.S/BS = osteoclast surface/bone surface; Tb = trabecula; bm = bone marrow.

We next investigated the role of Hfe in osteoclasts in relation to bone integrity. To this end, we crossed *Hfe* ^*flox*^ mice with mice carrying Cre under the control of LysM promoter, which deletes in multiple myeloid lineages, including in early osteoclast lineage cells.^40^, ^41^, ^42^, ^43^ *Hfe* ^*LysMCre(+)*^ mutant mice are characterized by unaltered levels of circulating iron and show no increased iron deposition in the liver or in the femur, as revealed by Prussian blue staining for iron on liver and femur sections (Fig. 2 *A, B*).^10^ Treatment of primary osteoclast precursors with soluble RANKL (50 ng/mL) for 7 days produced TRAP + multinucleated osteoclasts, irrespective of whether Hfe was present or not (Fig. 2 *C, D*). Regarding bone status, no statistically significant differences in the BV/TV, the Tb.N, the Tb.Th, and the Tb.Sp in the femur and the vertebra were detected between *Hfe* ^*LysMCre(+)*^ mutant and *Hfe* ^*LysMCre(−)*^ control littermates (Fig. 2 *E–G*). Likewise, no changes in the number of osteoblasts, osteoclasts, and osteocytes (Fig. 2 *H, I*) or in the serum levels of P1NP and CTX‐I markers (Fig. 2 *J*) were detected in *Hfe* ^*LysMCre(+)*^ mutant mice when compared with sex/age‐matched *Hfe* ^*LysMCre(−)*^ control littermates. Similarly, the analysis of major iron and bone parameters in aged *Hfe* ^*LysMCre(+)*^ mutant mice revealed no statistically significant difference with regard to control mice (Supplementary Fig. 2).

Collectively, our data imply that direct Hfe actions in osteoblasts and osteoclasts are dispensable for the maintenance of bone and iron homeostasis in mice under steady‐state conditions.

### Iron overload in two mouse models of Hfe hemochromatosis does not impair bone integrity

**Figure 3:**
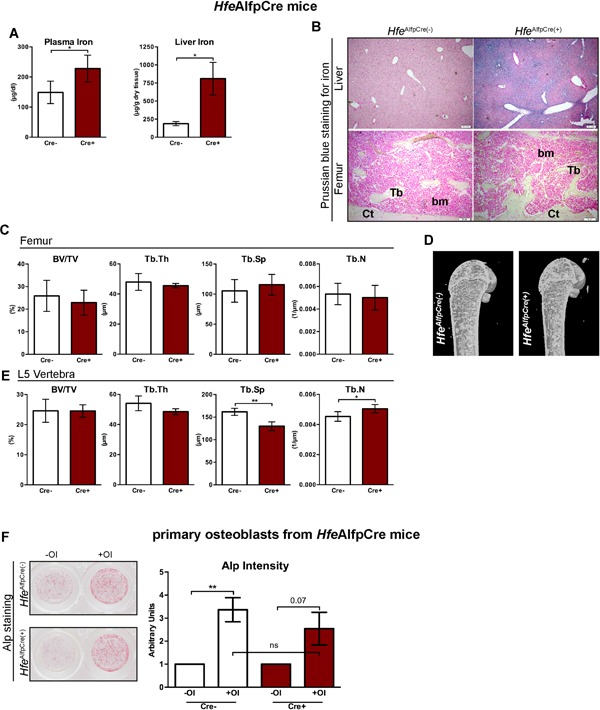
The presence of normal unaltered bone homeostasis in Hfe^AlfpCre^ mouse models of Hfe‐HH. (*A* **)** Iron levels in the blood and the liver in *Hfe* ^*AlfpCre(+)*^ and *Hfe* ^*AlfpCre(−)*^ mice (*n* = 4; 4). (*B*) Prussian blue staining for iron depositions in the liver and femoral bone (decalcified) of *Hfe* ^*AlfpCre(+)*^ and control mice. Images are representative staining of three mice per each group. The pictures represent 40× (liver) and 100× (bone) magnification. (*C–E*) µCT analysis of trabecular bone of distal femur and in the vertebra from *Hfe* ^*AlfpCre(+)*^ and *Hfe* ^*AlfpCre(−)*^ mice (*n* = 4; 6). (*D*) µCT 3D models of femora showing no evident changes in the composition of the trabecular bone, cortical bone, and marrow area. Images are representative of three mice per each group. (*F*) Alkaline phosphatase (ALP) staining and activity of primary osteoblasts from *Hfe* ^*AlfpCre(+)*^ and *Hfe* ^*AlfpCre(−)*^ mice (*n* = 7 to 8 per genotype) cultured in the presence or absence of osteoblast differentiation medium (OI) for 7 days. ALP staining was performed on day 6. Representative images of primary osteoblasts derived from calvarias of neonatal *Hfe* ^*Alfopre(+)*^ and *Hfe* ^*AlfpCre(−)*^ mice. ALP activity was measured in the supernatant at day 6 of osteoblast differentiation and was normalized to cell viability. Data were analyzed using GraphPad Prism software and results are shown as mean ± SD. For the statistical analysis, a nonparametric distribution and the Mann‐Whitney *U* test were used. *p values < .05, **p values < .01. All mice were males 13 weeks of age. BV/TV = bone volume/tissue volume; Tb.Sp = trabecular separation; Tb.Th = trabecular thickness; Tb.N = trabecular number; Tb = trabecular bone; Ct = cortical bone; bm = bone marrow; OI = osteogenic induction.

Subsequently, we tested whether *Hfe* ^*AlfpCre(+)*^ mutant mice show bone loss. In contrast to *Hfe* ^*Runx2Cre(+)*^ and *Hfe* ^*LysMCre(+)*^ mutant mice, *Hfe* ^*AlfpCre(+)*^ mutant mice develop systemic iron overload characterized by increased iron levels in the blood and the liver to an extent similar to that in mice with global *Hfe*‐deficiency (Fig. 3 *A, B*).^10^ However, no increased iron deposition was observed in either the trabeculas or in the marrow of long bones from *Hfe* ^*AlfpCre(+)*^ mutant mice (Fig. 3 *B*). Moreover, there were no significant changes in BV/TV, or in the thickness, number, and separation of the trabeculas in the femur, whereas statistically significant changes in the Tb.Sp and Tb.N were only detected in the vertebra of *Hfe* ^*AlfpCre(+)*^ mutant mice with regard to sex/age‐matched *Hfe* ^*AlfpCre(−)*^ control littermates (Fig. 3 *C–E*). The observed differences in Tb.Sp and Tb.N in vertebra were, however, not sufficient to result in overall changes in BV/TV (Fig. 3 *E*). Moreover, we showed that the differentiation capacity of primary osteoblasts, isolated from *Hfe* ^*AlfpCre(+)*^ mutant and control pups, was similar between cells (Fig. 3 *F*). These data suggest that a lack of hepatic *Hfe* and the subsequent systemic iron overload do not affect bone integrity.

**Figure 4:**
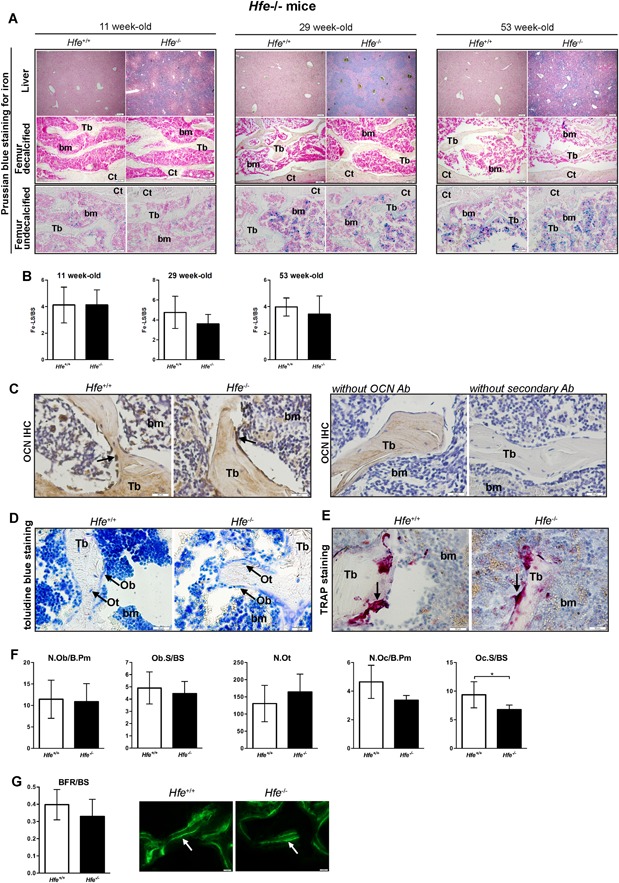
Intact bone integrity in aged male Hfe^−/−^ mice. (*A*) Prussian blue staining for iron depositions in the liver and femoral bone (decalcified and undecalcified) of young, middle‐aged, and aged *Hfe* ^−/−^ and control littermates. Images are representative staining of three mice per each group. The pictures represent 40× (liver) and 100× (bone) magnification. (*B*) The quantification of iron staining in the bones (expressed as iron‐labeled surface/bone surface [Fe‐LS/BS]). (*C*) Osteocalcin immunohistochemistry. Black arrows indicate positive osteoblasts. Images are representative staining of three mice per each group. The pictures represent 400× magnification. (*D*) Toluidine blue staining for osteoblasts and osteocytes in the femur. Specific cells in bone sections are indicated by an arrow. Images are representative staining of three mice per each group. The pictures represent 400× magnification. (*E*) TRAP‐positive cells in bone sections are indicated by an arrow and are stained red. Images are representative staining of three mice per each group. The pictures represent 400× magnification. (*F*) Histomorphometry showing no significant changes in osteoblast, osteocyte, and osteoclast numbers between *Hfe* ^−/−^ and *Hfe* ^*+/+*^ mice (*n* = 6; 5). (*G*) No changes in bone formation rate (BFR) between *Hfe* ^−/−^ and *Hfe* ^*+/+*^ mice (*n* = 6; 5) measured by calcein fluorochrome labeling. Images are representative staining of three mice per each group. Arrows indicate dual calcein incorporation. Data were analyzed using GraphPad Prism software and results are shown as mean ± SD. For the statistical analysis, a nonparametric distribution and the Mann‐Whitney *U* test were used. *p values < .05. N.Ob/B.Pm = osteoblast number/bone perimeter; Ob.S/BS = osteoblast surface/bone surface; N.Ot = osteocyte number; N.Oc/B.Pm = osteoclast number/bone perimeter; Oc.S/BS = osteoclast surface/bone surface; BFR/BS = bone formation rate/bone surface; Tb = trabecula; bm = bone marrow; Ob = osteoblasts; Ot = osteocytes.

Given this unexpected result, we reconsidered the mice with a global *Hfe* mutation, which is a well‐established mouse model of *Hfe*‐HH. In contrast to profound iron deposition in the liver of *Hfe* ^*−/−*^ mice (Fig. 4 *A*, Table 1), there was no increased iron accumulation in the marrow or in the trabeculas of long bones of both *Hfe* ^−/−^ and control *Hfe* ^+/+^ littermate mice at any age (Fig. 4 *A*). Surprisingly, the analysis of bone parameters, such as BV/TV, and the number, thickness, and separation of the trabeculas, in the femur and the vertebra, revealed no statistically significant differences between *Hfe* ^−/−^ and control mice (Table 1). These data reinterate the findings from the *Hfe* ^*AlfpCre(+)*^ mice and contradict the view that an excess of iron leads to bone loss in *Hfe*‐hemochromatosis mice.

**Table 1:** Iron and Bone Status in 11, 29, and 53 Weeks of Age Hfe^−/−^ Male Mice

Age	11‐weeks‐old		29‐weeks‐old		53‐weeks‐old	
Strain	*Hfe* ^+/+^	*Hfe* ^−/−^	*Hfe* ^+/+^	*Hfe* ^−/−^	*Hfe* ^+/+^	*Hfe* ^−/−^
*n* (males)	*6*	*4*	*6*	*7*	*5*	*7*
µCT Femur						
BV/TV (%)	*13.9* ± *3.4*	*16.2* ± *3.3*	*15.1* ± *4.3*	*15.4* ± *3.7*	*6.6* ± *3.0*	*6.3* ± *2.4*
Tb.Th (µm)	*62.8* ± *3.3*	*61.3* ± *2.1*	*74.6* ± *4.9*	*74.0* ± *7.8*	*72.1* ± *6.9*	*70.1* ± *7.0*
Tb.Sp (µm)	*249.2* ± *13.8*	*220.5* ± *31.1*	*249.1* ± *28.0*	*247.0* ± *28.9*	*343.4* ± *69.2*	*311.0* ± *47.9*
Tb.N (mm‐1)	*2.2* ± *0.4*	*2.7* ± *0.6*	*2.0* ± *0.5*	*2.1* ± *0.5*	*0.9* ± *0.4*	*0.9* ± *0.4*
Cs.Th (µm)	*211.8* ± *11.3*	*211.7* ± *6.6*	*216.9* ± *3.8*	*207.0* ± *4.9* **	*188.6* ± *14.9*	*188.5* ± *6.4*
µCT Vertebra						
BV/TV (%)	*27.2* ± *2.2*	*23.5* ± *2.2* *	*32.9* ± *4.3*	*34.8* ± *5.8*	*20.2* ± *6.3*	*23.0* ± *4.7*
Tb.Th (µm)	*65.2* ± *5.8*	*63.2* ± *1.8*	*75.4* ± *4.1*	*77.7* ± *7.5*	*64.3* ± *4.8*	*65.2* ± *2.7*
Tb.Sp (µm)	*185.3* ± *4.2*	*174.2* ± *12.0*	*288.7* ± *32.7*	*277.9* ± *28.5*	*228.8* ± *38.0*	*205.7* ± *32.8*
Tb.N (mm‐1)	*4.0* ± *0.2*	*4.3* ± *0.1*	*4.4* ± *0.4*	*4.5* ± *0.5*	*3.1* ± *0.8*	*3.5* ± *0.7*
Serum bone parameters						
P1NP (ng/mL)	*131.14* ± *41.96*	*107.19* ± *13.87*	*38.06* ± *10.6*	*39.95* ± *11.57*	*31.71* ± *5.71*	*35.19* ± *3.31*
CTX‐I (ng/mL)	*20.97* ± *2.66*	*13.18* ± *1.93* **	*12.34* ± *2.3*	*12.86* ± *2.4*	*11.53* ± *2.74*	*12.76* ± *1.5*
Testosterone (ng/mL)	*4.49* ± *5.52*	*1.96* ± *2.58*	*7.59* ± *5.81*	*6.12* ± *6.3*	*3.52* ± *3.68*	*2.75* ± *2.88*
Iron levels						
Plasma (µg/dL)	*133.6* ± *15.9*	*163.6* ± *14.6* *	*92.8* ± *14.1*	*151.3* ± *17.3* ***	*101.25* ± *19.0*	*149.3* ± *27.8* *
Liver (µg/g)	*178.5* ± *16.7*	*502.5* ± *84.6* **	*219.8* ± *77.5*	*615.7* ± *130.8* ***	*256.6* ± *131.3*	*679.9* ± *172.6* *
Spleen (µg/g)	*1183.5* ± *296.8*	*1054.1* ± *344.9*	*907.8* ± *298.8*	*915.6* ± *273.7*	*1257.5* ± *672.1*	*823.7* ± *559.0*
Duodenum (µg/g)	*346.2* ± *114.8*	*212.1* ± *59.2*	*256.2* ± *74.2*	*220.8* ± *72.1*	*266.7* ± *44.8*	*265.5* ± *36.8*
Pancreas (µg/g)	*103.72* ± *60.8*	*74.4* ± *28.9*	*n.d*	*n.d*	*n.d*	*n.d*
Hematological indices						
RBC (106/ mm3)	*8.05* ± *0.21*	*8.63* ± *0.11* ***	*8.88* ± *0.55*	*9.74* ± *0.56* *	*8.53* ± *0.34*	*8.5* ± *0.84*
Hgb (g/dL)	*15.35* ± *0.54*	*17.25* ± *0.33* ***	*14.93* ± *0.6*	*17.11* ± *0.87* ***	*14.4* ± *1.35*	*14.89* ± *2.02*
HCT (%)	*42.8* ± *1.4*	*48.62* ± *1.62* **	*46.68* ± *2.04*	*53.87* ± *3.12* ***	*44.25* ± *1.57*	*47.22* ± *4.65*
MCV (µm 3)	*53.17* ± *0.75*	*56.5* ± *1.29* *	*53* ± *1.21*	*55* ± *0.95* **	*53.0* ± *0.63*	*55.45* ± *1.81* ***
MCH (pg)	*19.08* ± *0.35*	*19.98* ± *0.21* **	*16.85* ± *0.47*	*17.57* ± *0.35* *	*16.85* ± *1.15*	*17.56* ± *2.3*
MCHC (g/dL)	*35.85* ± *0.36*	*35.48* ± *0.5*	*32* ± *0.36*	*31.81* ± *0.34*	*32.47* ± *2.41*	*31.65* ± *3.86*

Data were analyzed using GraphPad Prism software and results are shown as mean ± SD. For the statistical analysis, a nonparametric distribution and the Mann‐Whitney *U* test were used.

BV/TV = bone volume/tissue volume; Tb.Th = trabecular thickness; Tb.Sp = trabecular separation; Tb.N = trabecular number; Cs.Th = cortical thickness; P1NP = procollagen type 1 amino‐terminal propeptide; CTX‐I = C‐terminal telopeptide I; RBC = red blood cells; Hgb = hemoglobin; HCT = hematocrit; MCV = mean corpuscular volume; MCH = mean corpuscular hemoglobin; MCHC = mean corpuscular hemoglobin concentration; n.d. = no data available.

* *p* values <.05

** *p* values <.01

*** *p* values <.005.

### Gender‐ and age‐dependent bone mass are not affected in Hfe^−/−^ mice

**Figure 5:**
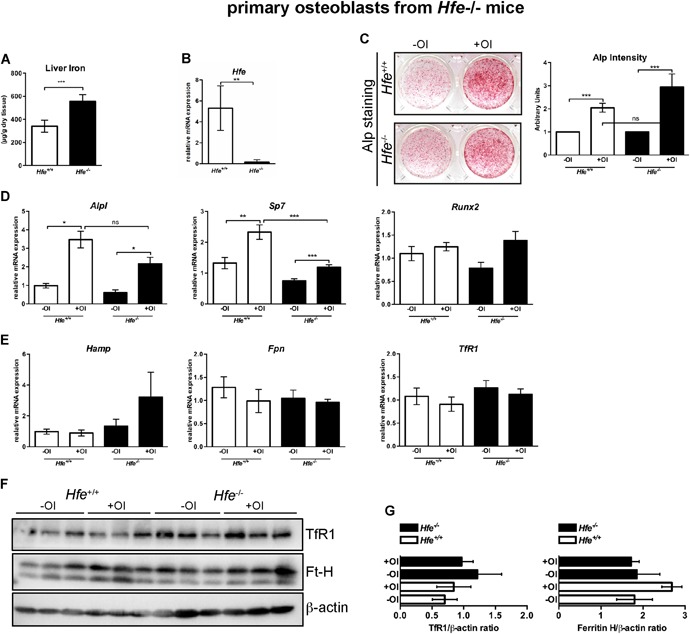
(*A*) Increase in nonheme iron content in the liver of 3‐ to 5‐day‐old *Hfe* ^−/−^ pups. (*B*) Relative mRNA expression of *Hfe* in primary osteoblasts from *Hfe* ^−/−^ and *Hfe* ^*+/+*^ mice, measured by quantitative real‐time PCR and calculated relative to the expression of the reference gene *Gapdh*. (*C*) Alkaline phosphatase (ALP) staining and its activity in primary osteoblasts from *Hfe* ^*−/−*^ and control mice cultured in the presence or absence of osteoblast differentiation medium (OI) for 7 days. ALP staining was performed on day 6. Representative images of primary osteoblasts derived from calvarias of neonatal *Hfe* ^*−/−*^ and *Hfe* ^*+/+*^ mice (*n* = 4 per genotype; each replicate was a pool of three to five mice). ALP activity was measured in the supernatant at day 6 of osteoblast differentiation and was normalized to cell viability. (*D, E*) Relative mRNA expression of genes involved in osteoblast differentiation (*Alp*, *Sp7*, *Runx2*) and of iron genes (*Hamp*, *Fpn*, *TfR1*) from primary osteoblasts of *Hfe* ^−/−^ and *Hfe* ^*+/+*^ mice, measured using quantitative real‐time PCR, and calculated relative to the expression of the reference gene *Gapdh*. (*F, G*) Immunoblot analysis of TfR1, ferritin H, and β‐actin protein levels in primary osteoblasts of *Hfe* ^−/−^ and *Hfe* ^*+/+*^ mice following differentiation and relative quantification using ImageJ software (NIH, Bethesda, MD, USA; https://imagej.nih.gov/ij/). Data were analyzed using GraphPad Prism software and results are shown as mean ± SD. For the statistical analysis, a nonparametric distribution and the Mann‐Whitney *U* test were used. *p values < .05, ***p values < .005. OI = osteogenic induction.

Our findings are highly surprising as previous studies reported bone loss in *Hfe* ^−/−^ mice.^31^, ^32^ Given that skeletal deterioration in humans and in mice may progress with advancing age,^44^ we next performed an in‐depth systematic analysis of major iron and bone parameters in aged *Hfe* ^−/−^ mice. We found that iron levels were consistently increased in the blood and liver of 29‐ and 53‐week‐old *Hfe* ^−/−^ mice (Table 1, Fig. 4 *A*); however, no excessive iron accumulation was detected in long bones (Fig. 4 *A, B*). Importantly, there was no statistically significant difference in the BV/TV, and the number, thickness, and separation of the trabeculas in the femur or in the vertebra of aged *Hfe* ^−/−^ mice with regard to control littermates (Table 1). Accordingly, the expression levels of serum P1NP and CTX‐I, markers of bone formation and bone resorption activity, respectively, as well as of testosterone, were not significantly different between aged *Hfe* ^−/−^ mutant and control littermate mice (Table 1). These observations were further supported by (1) histomorphometric analysis, which revealed no change in the number of osteoblasts, osteocytes, or osteoclasts in aged *Hfe* ^−/−^ mice with regard to control mice with the only exception in the osteoclast surface per bone surface parameter (Fig. 4 *C–F*); (2) the BFR measurements, which were comparable between aged *Hfe* ^−/−^ mice and control littermates (Fig. 4 *G*); moreover, we showed that (3) the differentiation capacity of primary osteoblasts, isolated from iron‐overloaded *Hfe* ^−/−^ and *Hfe* ^+/+^ control pups, was similar between cells (Fig. 5 *A–C*); and (4) the mRNA and protein levels of bone and iron markers (such as *Alp*, *Runx2*, *Hamp*, *Fpn*, *TfR1*, and *Ft‐H*) were comparable between *Hfe*‐deficient and *Hfe* ^+/+^ primary osteoblasts (Fig. 5 *D–G*). Of note, the mRNA expression of *Sp7* was significantly lower in differentiated osteoblasts isolated from *Hfe* ^−/−^ pups with regard to *Hfe* ^+/+^ control pups (Fig. 5 *D*). This was, however, the only gene whose changed mRNA expression was not sufficient enough to affect the overall differentiation capacity of primary osteoblasts, as can be seen by the measurement of ALP staining and ALP intensity in Fig. 5 *C*. Taken together, our findings contravene the previously proposed negative effects of iron overload on bone loss in aged *Hfe* ^−/−^ mice.

Given that the penetrance of *HFE/Hfe*‐HH varies between men and women and that in humans and in mice, the iron and the bone homeostasis are influenced by sex, diet, and genetic background,^45^, ^46^, ^47^, ^48^, ^49^ we also analyzed the bone status in female *Hfe* ^−/−^ mice at the ages of 11, 29, and 53 weeks, kept under the same conditions as their male counterparts. In sum, our data showed that *Hfe* ^−/−^ females displayed no signs of bone anomalies at any age studied despite the presence of systemic iron overload (Table 2). These findings led us to further conclude that gender‐ and age‐based differences do not suffice to induce bone‐associated pathologies previously described in *Hfe* ^−/−^ mice and in a subset of *HFE*‐HH patients.^27^, ^28^, ^29^, ^30^

**Table 2:** Iron and Bone Status in 11‐, 29‐, and 53‐Week‐Old Hfe^−/−^ Female Mice

Age	11 weeks old		29 weeks old		53 weeks old	
Strain	*Hfe* ^+/+^	*Hfe* ^−/−^	*Hfe* ^+/+^	*Hfe* ^−/−^	*Hfe* ^+/+^	*Hfe* ^−/−^
*n* (females)	*7*	*4*	*7*	*7*	*8*	*9*
µCT Femur						
BV/TV (%)	*5.2* ± *2.6*	*5.8* ± *2.0*	*2.5* ± *0.8*	*3.6* ± *1.5*	*1.0* ± *0.6*	*1.3* ± *0.6*
Tb.Th (µm)	*54.7* ± *2.4*	*59.2* ± *4.2*	*58.3* ± *8.7*	*58.0* ± *9.2*	*62.1* ± *15.7*	*65.7* ± *11.9*
Tb.Sp (µm)	*315.2* ± *19.7*	*340.0* ± *26.1*	*384.8* ± *23.4*	*343.8* ± *32.6* *	*586.1* ± *102.2*	*522.0* ± *58.9*
Tb.N (mm‐1)	*0.9* ± *0.5*	*1.0* ± *0.3*	*0.4* ± *0.1*	*0.6* ± *0.2*	*0.2* ± *0.1*	*0.2* ± *0.1*
Cs.Th (µm)	*186.8* ± *7.8*	*187.2* ± *7.1*	*221.5* ± *8.7*	*220.0* ± *11.8*	*200.1* ± *13.7*	*204.5* ± *9.0*
µCT Vertebra						
BV/TV (%)	*14.9* ± *2.3*	*15.1* ± *0.6*	*22.5* ± *3.6*	*25.2* ± *2.0*	*19.7* ± *4.4*	*22.4* ± *3.4*
Tb.Th (µm)	*49.1* ± *2.7*	*50.5* ± *1.5*	*73.3* ± *3.2*	*77.7* ± *4.3*	*74.3* ± *4.4*	*74.1* ± *3.8*
Tb.Sp (µm)	*224.7* ± *17.5*	*234.0* ± *14.4*	*288.7* ± *32.7*	*277.9* ± *28.5*	*331.2* ± *62.7*	*334.9* ± *56.2*
Tb.N (mm‐1)	*3.0* ± *0.4*	*3.0* ± *0.1*	*3.1* ± *0.5*	*3.3* ± *0.3*	*2.6* ± *0.5*	*3.0* ± *0.5*
Serum Bone Parameters						
P1NP (ng/mL)	*130.3* ± *40.6*	*78.1* ± *12.4* **	*29.4* ± *8.3*	*33.2* ± *4.3*	*33.9* ± *5.6*	*38.5* ± *7.2*
CTX‐I (ng/mL)	*21.7* ± *4.1*	*22.3* ± *6.7*	*12.2* ± *2.3*	*12.7* ± *2.8*	*12.0* ± *3.9*	*14.0* ± *2.1*
Iron levels						
Plasma (µg/dL)	*140.7* ± *26.5*	*182.8* ± *24.8* *	*105.6* ± *26.3*	*166.2* ± *39.0* *	*118.7* ± *31.7*	*166.4* ± *24.4* *
Liver (µg/g)	*214.8* ± *45.2*	*942.7* ± *130.9* **	*345.2* ± *82.2*	*999.9* ± *220.5* **	*555.7* ± *269.3*	*1041.6* ± *127.8* **
Spleen (µg/g)	*1800.0* ± *443.3*	*836.8* ± *177.6* **	*1123.2* ± *255.7*	*875.8* ± *349.2 (0.07)*	*1262.57* ± *401.57*	*847.0* ± *346.05* *
Duodenum (µg/g)	*284.0* ± *133.7*	*201.6* ± *99.9*	*272.5* ± *46.5*	*240.6* ± *58.7*	*317.8* ± *87.7*	*324.5* ± *112.8*
Pancreas (µg/g)	*103.7* ± *60.8*	*74.4* ± *28.9*	*n.d*.	*n.d*.	*123.7* ± *30.3*	*150.2* ± *34.5*
Hematological indices						
RBC (106/ mm3)	*7.8* ± *0.3*	*8.7* ± *0.4* **	*8.9* ± *0.9*	*9.5* ± *0.4* *	*8.5* ± *0.4*	*7.7* ± *0.5*
Hgb (g/dL)	*15.5* ± *0.5*	*17.2* ± *0.6* *	*15.0* ± *1.1*	*17.0* ± *0.6* **	*15.7* ± *0.9*	*15.9* ± *0.8*
HCT (%)	*41.9* ± *1.0*	*48.5* ± *1.9* **	*47.6* ± *1.8*	*52.5* ± *1.7* **	*44.8* ± *2.6*	*42.9* ± *2.5*
MCV (µm3)	*53.9* ± *1.5*	*55.5* ± *0.6*	*52.0* ± *1.2*	*55.0* ± *1.0* **	*53.0* ± *2.3*	*55.6* ± *2.1*
MCH (pg)	*19.9* ± *0.6*	*19.7* ± *0.3*	*16.7* ± *0.5*	*17.8* ± *0.4* **	*18.6* ± *0.9*	*20.7* ± *1.0* *
MCHC (g/dL)	*37.0* ± *0.5*	*35.4* ± *0.3* **	*32.4* ± *0.4*	*32.3* ± *0.5*	*35.1* ± *0.7*	*37.2* ± *2.4*

Data were analyzed using GraphPad Prism software and results are shown as mean ± SD. For the statistical analysis, a nonparametric distribution and the Mann‐Whitney *U* test were used.

BV/TV = bone volume/tissue volume; Tb.Th = trabecular thickness; Tb.Sp = trabecular separation; Tb.N = trabecular number; Cs.Th = cortical thickness; P1NP = procollagen type 1 amino‐terminal propeptide; CTX‐I = C‐terminal telopeptide I; RBC = red blood cells; Hgb = hemoglobin; HCT = hematocrit; MCV = mean corpuscular volume; MCH = mean corpuscular hemoglobin; MCHC = mean corpuscular hemoglobin concentration; n.d. = no data available.

* *p* values <.05.

** *p* values <.01.

## Discussion

Using three conditional *Hfe* knockouts and a global *Hfe* mutant mouse line, we separated, for the first time, the contribution of iron overload from the role of Hfe in osteoblasts and osteoclasts on bone integrity. We unequivocally show that systemic iron overload, at the degree present in *Hfe* ^−/−^ mice, does not associate with a microarchitecture impairment of long bones, thereby excluding both a direct effect of iron overload and a specific role for Hfe on bone integrity. Several lines of evidence support these conclusions: (1) no bone loss was present in *Hfe* ^−/−^ mice: young, middle‐aged, and aged male, as well as female *Hfe* ^−/−^ mice were all protected, despite systemic iron overload; (2) no bone loss was observed in liver‐specific *Hfe*‐mutant mice—which phenocopy the iron pathology of constitutive *Hfe* ^−/−^ mice—thus, iron overload, present in constitutive and liver‐specific *Hfe* mutant mice, does not disturb bone homeostasis; and finally, (3) no bone loss was present in mice lacking *Hfe* selectively in osteoblasts and osteoclasts, implying that Hfe actions in bone cells were not required to maintain systemic bone or iron homeostasis.

An initial investigation by Guggenbuhl and colleagues proposed iron overload and increased number of osteoclasts as the main cause of bone loss in 6‐month‐old male *Hfe* ^−/−^ mice,^31^ a finding that was not confirmed in their follow‐up study.^32^ The latter showed a decrease in BFR in *Hfe* ^−/−^ mice and proposed for a negative effect of iron overload on the osteoblasts.^32^ Our study contradicts these findings. Mice of the same age (6‐ and 12‐month‐old mice), background (C57BL6), and sex (male mice) were used by Guggenbuhl and colleagues and by us, yet the incidence of bone loss was completely undetectable in our cohort. Our data argue against the negative effect of iron overload on osteoclasts because the number and the function of osteoclasts remained equal between *Hfe* ^−/−^ mutant and control mice (Fig. 4 *E, F*). Furthermore, no effect of iron overload was observed on osteoblasts, as the serum P1NP levels, number of osteoblasts, and BRF were comparable between *Hfe* ^−/−^ and control mice (Table 1). Moreover, there was no difference in the differentiation capacity of primary osteoblasts isolated from constitutive *Hfe* ^−/−^ or from *Hfe* ^*AlfpCre(+)*^ mice (Figs. 3 *F*, 5 *C*). Finally, the levels of iron in the diet seem to be comparable between the studies excluding iron as the major factor contributing to differences between the data. We suggest that the discrepancy between our data with regard to bone phenotype might be explained by the use of nonlittermate mice as controls in an initial report by Guggenbuhl and colleagues. From a number of studies, it is known that differences in using nonlittermates can account for a variation in bone phenotype.^50^, ^51^ We are therefore confident that using control littermates safeguards the accuracy of our analyses.

If our study refutes any negative effect of iron overload on bone loss in *Hfe* ^−/−^mice, why then is iron still considered as the guilty one? The hypothesis that iron acts as the underlying cause of bone abnormalities in iron‐overloaded conditions has emerged from the role of free, nonbound iron, which is present when iron‐binding capacities of plasma transferrin or intracellular iron‐storage molecule ferritin are surpassed. Several in vitro and in vivo studies have shown that changes in intracellular catalytically active iron levels may play a critical role in inducing oxidative stress and triggering pathways connected with inflammatory processes, which in turn may affect bone metabolism. For example, a study by Tsay and colleagues showed that parenteral iron injections produced severe iron overload in bones and that the degree of iron overload correlated with an increase in osteoclast number, causing changes in bone microarchitecture and bone loss.^52^ At the molecular level, iron excess induced oxidative stress via activation of the NF‐κB pathway and the production of proinflammatory cytokines, which in turn led to bone loss through increased osteoclastogenesis.^52^, ^53^ Conversely, treatment of mice with N‐acetyl‐L‐cysteine prevented iron‐induced bone anomalies.^52^ However, injections of a 10‐times lower dose of iron‐dextran^52^ or of ferric ammonium citrate,^54^ which resulted in severe systemic iron overload, had minimal effect on bone loss. Our study shows that iron overload, at the levels present in mouse models of genetic *Hfe*‐hemochromatosis (*Hfe* ^−/−^ and *Hfe* ^*AlfpCre(+)*^ mice), does not suffice to induce bone loss under steady‐state conditions. We suspect that the degree of iron‐loading matters, which may be a critical factor to trigger, or to contribute to, the development of osteoporosis in mice. However, there are several considerations to be taken into account when comparing *Hfe*‐HH and other models of acquired iron‐overload disorders. First, the etiology of iron overload in *Hfe*‐HH is genetic, that is caused by the lack of *Hfe* and consequently low hepatic hepcidin production.^10^ Parenteral iron injections, on the other hand, result in increased hepcidin expression.^15^ Next, the pattern of iron overload in terms of iron distribution in the bones is different between iron‐injected and *Hfe* ^−/−^ mice. Whereas heavy iron deposits were present in the marrow and the trabeculas of iron‐injected mice,^52^ no increased iron deposition was observed in the femur of *Hfe* ^−/−^ mice at any age analyzed (Fig. 4 *A, B*). Despite having “iron” as a common denominator, these iron disorders are profoundly different; thus, bone‐related pathologies cannot be easily compared with one another.

Based on our data from *Hfe* ^−/−^ mice, and the still limited number of clinical observations in *HFE* patients,^27^, ^28^, ^29^, ^30^ we believe that osteoporosis does not primarily arise from iron overload. We suggest that potential triggers throughout the course of the disease progression could induce malfunction in bones. A more simple explanation would be gonadal deficiency, a commonly known cause of bone loss, as the primary cause because of its co‐occurrence with iron overload in *HFE/Hfe*‐hemochromatosis. Whether iron overload in *Hfe* ^−/−^ mice may act as an additional culprit when other osteoporosis triggers are present, such as vitamin D deficiency, liver cirrhosis, and endocrinological defects, is currently unknown and interesting to investigate further. Under physiological steady‐state conditions, however, systemic iron overload in *Hfe‐*HH mice does not suffice to cause bone loss.

We are confident that our findings are of utmost relevance for the clinical care of *HFE*‐HH patients and will aid in targeting the mechanisms of bone loss during iron‐overload pathology rather than focusing on the diversion of *HFE/Hfe* mutations and the iron overload itself.

## Disclosures

The authors declare no conflict of interest.

## Supporting information

Supporting information.
