# Supplementary material for: Despite Genetic Iron Overload, Hfe‐Hemochromatosis Mice Do Not Show Bone Loss
Source: JBMR Plus. 2019 Jul 26;3(9):e10206. doi: 10.1002/jbm4.10206 (PMC6808227; doi:10.1002/jbm4.10206)

SUPPORTING FIGURE LEGENDS

Supporting Fig. 1. Hfe actions in osteoblasts are dispensable for the regulation of bone and iron metabolism in 29-weeks old $Hfe^{Runx2Cre}$ mice. (*A*) Recombination efficacy of *Hfe* in tissues of $Hfe^{Runx2Cre(+)}$ mice. Genomic DNA from $Hfe^{Runx2Cre(+)}$ mutant mice was isolated from indicated organs and *Hfe* allele was detected using PCR method. The upper band corresponds to the recombined *Hfe* allele (ko/ko), the middle to the floxed allele (f/f) and the lower to the wildtype allele (wt/wt); Cre(+): $Hfe^{Runx2Cre(+)}$ mutant mice. (*B, C*) Micro-CT analysis of trabecular bone at distal femur and in the vertebra of $Hfe^{Runx2Cre(+)}$ and $Hfe^{Runx2Cre(-)}$ mice (n=5; 4). *(D)* Histomorphometry showing no significant changes in osteoblast, osteocyte and osteoclast numbers between $Hfe^{Runx2Cre(+)}$ and $Hfe^{Runx2Cre(-)}$ mice (n=6; 5). (*E*) The expression levels of bone formation marker procollagen type 1 amino-terminal propeptide (P1NP), bone resorption marker C-terminal telopeptide I (CTX-I) in the serum of $Hfe^{Runx2Cre(+)}$ and $Hfe^{Runx2Cre(-)}$ mice (n=6; 6). (*F*) Circulating iron levels and the non-heme liver iron content in $Hfe^{Runx2Cre(+)}$ and $Hfe^{Runx2Cre(-)}$ mice (n=5; 4).

Data were analyzed using GraphPad Prism software and results are shown as mean ± SD (standard deviation). For the statistical analysis, a non-parametric distribution and the Mann-Whitney-U Test were used. * p values <.05, ** p values <.01. All mice were males 29-weeks of age.

BV/TV: bone volume/tissue volume; Tb.Sp: trabecular separation; Tb.Th: trabecular thickness; Tb.N: trabecular number.

Supporting Fig. 2. Hfe actions in osteoclasts are nonessential for the regulation of iron and bone metabolism in aged mice. (*A*) Iron levels in the blood and the liver of $Hfe^{LysMCre(+)}$ and $Hfe^{LysMCre(-)}$ mice (n=5; 6). (*B*) Micro-CT analysis of trabecular bone at distal femur of $Hfe^{LysMCre(+)}$ and $Hfe^{LysMCre(-)}$ mice (n=5; 6).

Data were analyzed using GraphPad Prism software and results are shown as mean ± SD (standard deviation). For the statistical analysis, a non-parametric distribution and the Mann-Whitney-U Test were used. * p values <.05. All mice were males 47-weeks of age.

BV/TV: bone volume/tissue volume; Tb.Sp: trabecular separation; Tb.Th: trabecular thickness; Tb.N: trabecular number.

Supporting Fig. 1

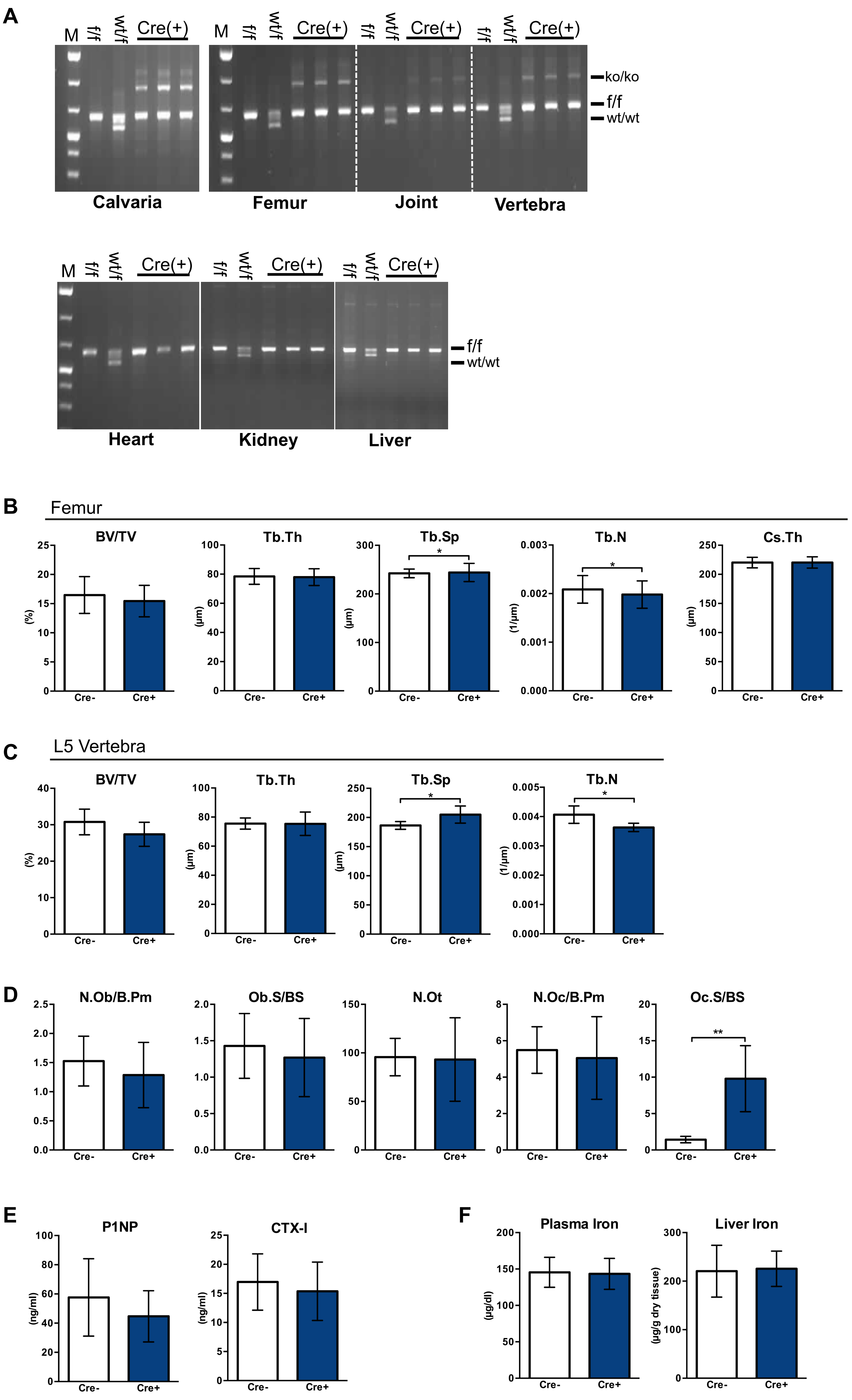

Supporting Fig. 2

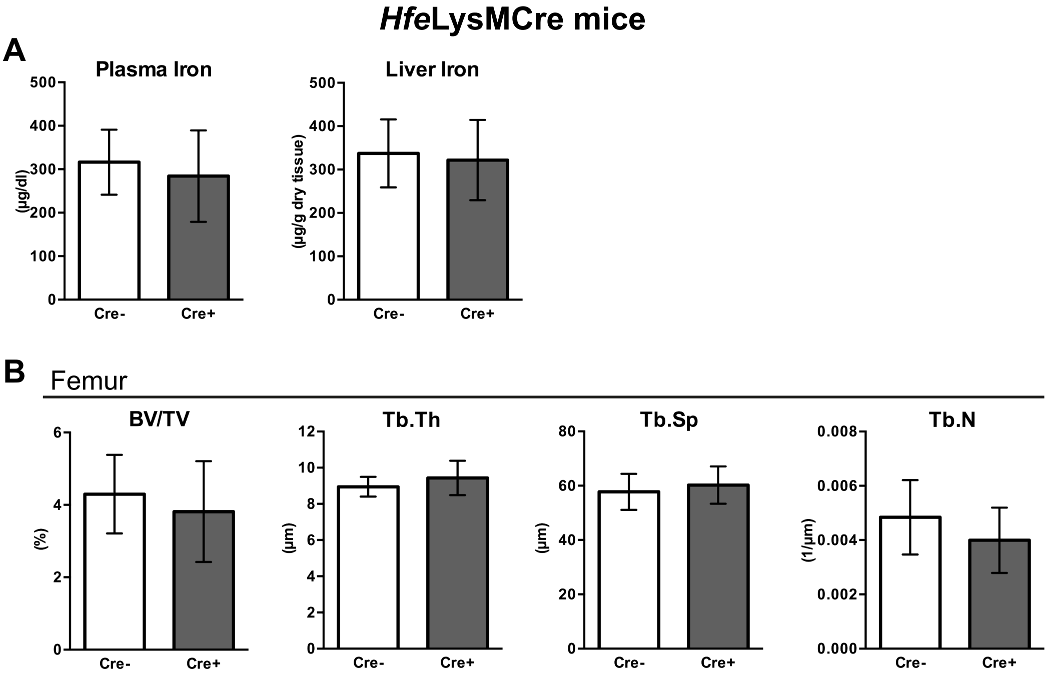

Supplement: Supplementary file 1 — Supporting information. [file JBM4-3-na-s001.pdf]
